# Longitudinal follow-up of mixed connective tissue disease and overlapping autoimmune diseases of childhood onset in the Afro-descendant population of the French West Indies

**DOI:** 10.1186/s12969-023-00951-3

**Published:** 2024-01-11

**Authors:** Arthur Felix, Lindsay Osei, Frederique Delion, Benoit Suzon, Aurore Abel, Moustapha Drame, Yves Hatchuel, Christophe Deligny, Fabienne Louis-Sidney

**Affiliations:** 1 Department of General Pediatrics, Competence Centre for Rheumatic, Autoimmune and Systemic Diseases in Children (RAISE) Antilles-GuyaneEpiCliV Research Unit, University of the French West Indies, Martinique University Hospital, Fort-de France, France; 2 Department of Pediatrics, Andrée Rosemon Hospital, Cayenne, France; 3 Department of Pediatrics, Guadeloupe University Hospital, Pointe-à-Pitre, France; 4 Department of Internal Medicine, Martinique University Hospital, Fort-de-France, France; 5 Department of Clinical Research and Innovation, Martinique University Hospital, Fort-de-France, France; 6 Department of Rheumatology, Martinique University Hospital, Fort-de-France, France; 7 MFME, CHU de la Martinique La Meynard, Fort-de-France, 97261 France

**Keywords:** Juvenile mixed connective tissue disease, Overlapping autoimmune disease autoimmune disease, Afro-Caribbean children, African ancestry children

## Abstract

**Introduction:**

Overlap autoimmune syndromes (OAS) and mixed connective tissue disease (MCTD) are rare in children. We performed a retrospective, longitudinal and descriptive study of Afro-Caribbean patients from the French West Indies followed for MCTD and OAS to describe their characteristics and outcomes during childhood.

**Methods:**

Retrospective study from January 2000 to 2023. Listings of patients were obtained from multiple sources: computerized hospital archives and national hospital-based surveillance system, registry of pediatricians and adult specialists in internal medicine and the national registry for rare diseases. MCTD was defined according to Kasukawa’s criteria. OAS was defined as overlapping features of systemic lupus erythematosus (SLE), systemic sclerosis (SSc), and dermatomyositis/autoimmune myositis (DM/AM).

**Results:**

Sixteen patients were included over a 23-year period (10 MCTD and 6 OAS). The incidence was 0.23 per 100,000 children-years. The mean age at diagnosis was 11.9 years old (2.4–17) with median follow up of 7.9 years (2.1–19.6). SLE phenotype was present in the highest, followed by SSc and DM/AM. Patients had an average of three flares during childhood (1–7). A quarter (25%) had symptomatic pulmonary arterial hypertension (PAH). Ninety-four percent received steroids during follow-up and 88% required a corticosteroid-sparing therapy. Three patients (19%) developed SLE after more than 10y of follow-up. There were no death and no chronic organ failure.

**Conclusion:**

This is the largest pediatric cohort of MCTD and OAS in Afro-descendant patients treated in a country with a high standard of care. The clinical evolution did not differ between MCTD and OAS. The main complication was PAH, more frequent in our cohort.

**Supplementary Information:**

The online version contains supplementary material available at 10.1186/s12969-023-00951-3.

## Key messages

1. First cohort of Afro-Caribbean pediatric patients with overlap autoimmune syndromes (OAS) and pediatric mixed connective tissue disease (MCTD)

2. The clinical phenotypes were predominantly systemic lupus and systemic sclerosis.

3. The most common comorbid complication was pulmonary arterial hypertension

4. The overall prognosis during follow-up was good and similar to European and American studies.

**Supplementary Information:**

The online version contains supplementary material available at 10.1186/s12969-023-00951-3.

## Introduction

Overlap autoimmune syndromes (OAS) are rare rheumatic diseases in children at the intersection of several systemic autoimmune diseases [1]. Mixed connective tissue disease (MCTD) is a distinct subset of overlap autoimmune syndromes characterised by overlapping features of systemic lupus erythematosus (SLE), juvenile idiopathic arthritis (JIA), systemic sclerosis (SSc), Gougerot-Sjögren syndrome (GSS) and dermatomyositis/autoimmune myositis (DM/AM) [2, 3]. The presence of high titers of anti-U1 ribonucleoprotein (U1-RNP) antibodies is a characteristic feature of MCTD [4]. The disease has a juvenile onset in up to 25% of cases [5]. Since it was first defined in the 1970s, there has been debate as to whether MCTD is a distinct entity among overlap syndromes. In populations of African ancestry, the clinical presentation and epidemiology of systemic auto-immune diseases, particularly connective tissue disease are not always similar to data from Caucasian or Asian populations [6–9]. The epidemiology and clinical features of MCTD and OAS in populations of African ancestry have not been well described heretofore, except in small series of adults from Gabon, Senegal and Ivory Coast [10–12]. There has never been a specific description in children of this ethnic group of African ancestry or in Afro-Caribbean population. It remains unclear whether this reported low incidence of the disease is due to genetic differences, access to care and the general level of health care in those countries or other unknown associated factors. The French West Indies (FWI, i.e. Martinique, Guadeloupe, and French Guiana) have a combined population of approximately 300,000 children under the age of 18 years [13]. The healthcare system is free and universal, with two university hospitals and reference centers approved by the French Ministry of Health. Although there are no official ethnicity statistics, a large majority of patients are of black African ancestry [14] (> 90%). The objectives of this study were to perform a retrospective, longitudinal and descriptive study of pediatric patients from the FWI followed-up for MCTD and OAS. We aimed to describe their clinical, immunological characteristics and outcomes during childhood.

## Methods

This was a retrospective study covering the period from January 2000 to January 2023. The methods used to identify patients aimed to cross-reference different sources to ensure exhaustive identification of all patients. In each reference center of the FWI, we searched the local registries of pediatric patients followed-up for OAS and MCTD by the referring pediatricians. We also extracted lists of patients from the electronic hospital archives and the French Medicalization of Information Systems Program (PMSI), which is a comprehensive national database containing all hospital discharge records, using the coded diagnosis of OAS and MCTD (M35, M350, M351, M358 and M359). We also extracted the list of patients registered in the electronic French national registry for rare diseases (BAMARA), a secure national information system. Subsequently, the lists of patients were analyzed for the relevance of the diagnoses, to check inclusion criteria and to eliminate duplicates. Kasukawa’s criteria [15] were chosen for the diagnosis of MCTD because they have been used in most published studies in children. We also took into consideration the criteria defined by Sharp and Alarcon-Sergovia [16, 17]. The patients with OAS had the features of more than two autoimmune diseases but did not meet the diagnostic criteria for MCTD. The diagnosis of SLE, SSc, GSS, DM/AM, was based on the American College of Rheumatology (ACR) diagnostic criteria for each disease [18–21]. Data collection was completed by reviewing follow-up and hospitalisation reports available in the patients’ computerised medical records using a standardised template. Paper medical records were also consulted to record follow-up data. Details of drug prescriptions were analysed using computerised prescriptions and manual prescriptions in the files. A clinical flare was defined as a clinical manifestation that required a change in background therapy or steroid pulse (> 1 mg/kg/day). Patients were included if they were born, grew up and lived in the FWI, while those not living in the FWI were excluded. The results were reported according to the STROBE methodology [22]. The Institutional Review Board of the University Hospital of Martinique approved the study under the number 2021/116.

## Results

**Fig. 1:**
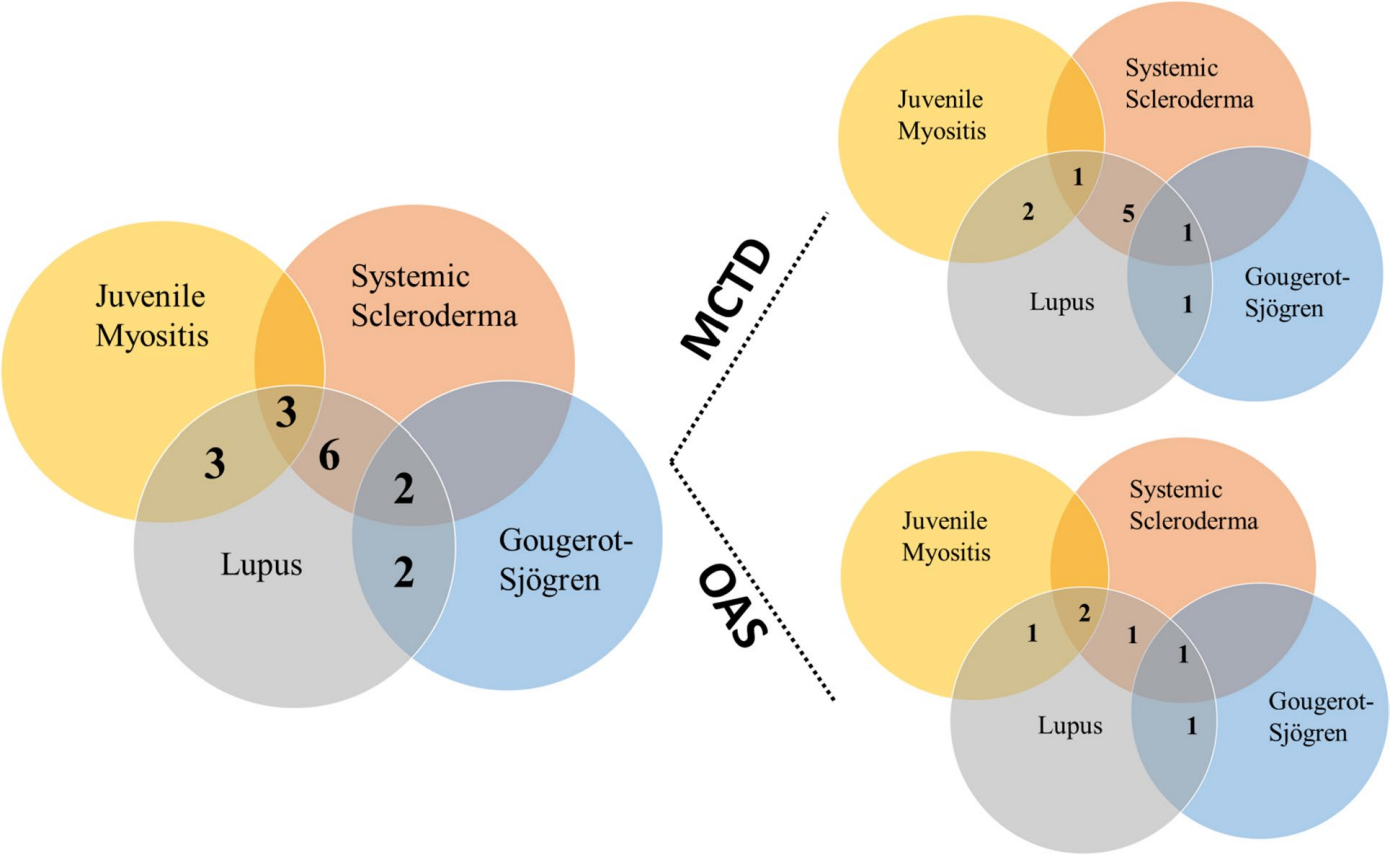
Clinical phenotypes and various combinations and interrelationships between the phenotypes at onset for pediatric patients with MTCD and OAS. The phenotypes included the clinical signs according to the ACR classifications of diseases (SLE, SSc, GSS, DM/AM) without taking into account the immunological/autoantibody features

Sixteen patients were included over a 23-year period. Ten patients fulfilled the Kasukawa criteria for the diagnosis of MCTD and at least one of the other criteria (Sharp or Alarcon-Sergovia). Six patients did not meet these criteria and were considered to have OAS. The flowchart of this study is shown in Supplementary Fig. 1. The combined incidence of OAS and MCTD observed in this study was estimated to be 0.23 per 100,000 children-years. There was a large majority of girls (88%, sex ratio 7:1). Their clinical and biological characteristics are summarized in Table 1. The mean age at diagnosis was 11.9 years old (2.4–17). The median follow-up was 7.9 years (2.1–19.6). The clinical phenotypes and the various combinations and interrelationships between the phenotypes at disease onset are shown in Fig. 1. Four patients (25%) had a flare-up of symptomatic pulmonary arterial hypertension (PAH) and were managed in the intensive care unit at some point. Of these 4 patients, 3 had PAH at diagnosis and one within the first year of follow-up. Patients with serositis (4/16, 25%) all had both pericarditis and pleural effusion.

**Table 1:** Patients characteristics with MCTD and OAS

**Clinical features**	**Results**
***N* = 16 patients**	
Median Age (years)	11.9 (2.4–17)
Median Follow-up (years)	7.9 (2.1–19.6)
Girls/Boys %	88/12 (7/1)
Family history of autoimmune disease %	31
Cutaneous manifestation %	94
Raynaud’s phenomenon %	44
Swollen fingers or hands %	62.5
Articular manifestation (arthalgia) %	88
Arthritis %	75
Mucosal manifestations ( oral ulcers) %	69
Fever %	69
Proximal muscle weakness and increased muscle enzymes %	25
Pulmonary manifestations	31
Serositis	25
Gastrointestinal manifestations	25
Pulmonary hypertension	25
Renal manifestations	20
Hepatic manifestations	12.5
Endocrine manifestations	12.5
Neurological manifestations	0
**Biological features**	
Anemia (Hb < 12 g/dl) %	56
Thrombocytopenia (< 100,000/mm3) %	50
Hypergammaglobulinemia %	75
ESR (mean value, mm after 1 h) %	70
CRP (mean value, mg/L) %	39
**Immunological profile**	
Anti nuclear antibody > 1/1280%	100
Anti-U1-RNP %	62.5
Anti-dsDNA %	56
Anti-SSA/ Anti-SSB %	44
Anti-Sm %	30
Biological APL %	20
Low C3/C4	44

The endocrine manifestations were the discovery of type 1 diabetes and autoimmune thyroiditis. Renal manifestations were lupus nephritis: 2 class III/IV and 1 class I/II

Pulmonary manifestations are restrictive pattern (reduce lung volumes and DLCO). Gastrointestinal manifestations are dysphagia and gastroesophageal reflux

Patients had an average of three flares during childhood (1–7). The majority of patients received hydroxychloroquine and steroids at least once during follow-up (94%). Fourteen (87.5%) required a corticosteroid-sparing therapy. Eight patients received Mycophenolate mofetil (50%) and six patients (38%) received methotrexate mainly for joint flares. Two patients received rituximab (12.5%) mainly for renal involvement. All patients were receiving background treatment and low-dose corticosteroids (2.5–5 mg per day) at the last follow-up.

Three patients (19%, 2 in the MCTD group and 1 in the OAS group) progressed to formal SLE criteria (additional immunological findings such as Anti-Sm +, lupus nephritis) after respectively 11, 12 and 13 years of follow-up respectively. There were no deaths in the cohort and no chronic organ failure (renal, cardiac or respiratory).

## Discussion

This retrospective study of the FWI over a period of 23 years identified a cohort of 16 patients with MCTD and OAS. This represents the largest cohort of pediatric patients of Afro-Caribbean and Black African ancestry reported with these diseases and with longitudinal follow-up data. The rarity of the description of these rare diseases in predominantly Afro-descendant populations is multifactorial. First, there are inequalities in the level of and access to health care in sub-Saharan Africa. Second, in the North American population, African American patients have historically experienced socioeconomic disparities that have negatively impacted access to care and participation in research studies [23, 24]. Third, African and Afro-descendant cultural belief systems around the mystical nature of autoimmune diseases may have influenced access to care and reporting of diagnoses [25]. Fourth, these patients may have been misdiagnosed and considered as SLE.

The age at diagnosis in our population was similar to previous pediatric series [5, 26]. Patients with MCTD and OAS in our cohort were predominantly female, with a female to male ratio of 7:1 which is consistent with previous reports [5]. Interestingly, swollen fingers or hands were more common in our cohort than Raynaud’s phenomenon. This manifestation is at the forefront of the disease and is often part of the initial presentation in Caucasian patients as well as in other series reported in India [26–28], Saudi Arabia and China with more than 80% of patients having Raynaud’s phenomenon [29–31]. Raynaud’s phenomenon appears to be rare in the MCTD of black African subjects [10–12]. There were only two cases of Raynaud’s phenomenon among the 10 patients in the series from Gabon and Senegal [10, 11]. However, given the climate of the Caribbean or sub-Saharan Africa, it is also possible that the incidence of this syndrome is underestimated in the complaints received because of its lesser impact on quality of life [32]. The proportion of serositis was comparable to published adult series [5, 33]. In our cohort, the SLE-like phenotype was present in the highest frequency of patients, followed by SSc and AM/PM-like phenotypes, which has already been described in the Caucasian cohort [3, 34]. Overall, apart from some nuances, there does not seem to be any particular pattern in terms of clinical or biological manifestations in our pediatric cohort of patients of African ancestry.

The incidence of MCTD and OAS observed in our study was similar and consistent compared with the previous studies in Caucasian populations [27, 35]. This is not the case for other connective tissue diseases in the Afro-Caribbean population of the FWI where the described incidence is higher than in Caucasian populations [7, 8]. This incidence is consistent with data from American series showing a low proportion of African-Americans in MCTD studies [28], much lower than in studies about systemic lupus [36]. In our series, the clinical symptoms, organ involvement and clinical or biological changes during follow-up did not differ significantly between MCTD and OAS patients. However, formal conclusions could not be drawn due to the limited size of our study. This is in contrast to larger Turkish or French studies that showed different clinical presentations and outcomes between MCTD and OAS [33, 37]. However, our small number of patients did not allow us to conclude on an African ethnic character that makes the phenotypes and evolutionary profile specific. We found no mortality over a 23-year period. Mortality in previous MCTD in pediatric population was low but not null [34, 38]. However, it remains difficult to compare the outcome of MCTD in different cohorts. This is mainly because treatments and definitions of disease remission are not uniform and long-term follow-up studies are scarce. In the predominantly Caucasian Norwegian, American and French cohorts, more than two thirds of patients were considered to have active disease at their last visit [3, 26, 34]. However, our analysis may have been limited by our small sample size.

The existence of MCTD as a distinct clinical entity has been debated. Previous studies suggested that the majority of patients with MCTD would progress to other connective tissue diseases, most commonly SLE and SSc [39]. However, more recent studies showed a different result, as evolution was observed in less than one out of 5 patients [5, 33]. Our results were more consistent with these studies, as evolution to pure systemic lupus was observed for only three patients (19%) after more than 10 years of follow-up.

In terms of treatment, there is currently no consensus on a recommended treatment regimen for MCTD or OAS. Most patients in our series required an immunosuppressive drug and more than 90% received corticosteroids or hydroxychloroquine during follow-up. This is consistent with pediatric cohorts with longitudinal follow-up, which are predominantly Caucasian [5, 26, 34]. As the main phenotype was SLE in our cohort, the therapies were influenced by the treatment of this disease. The overall prognosis of our patients was good, with no deaths and no organ failure, although a high percentage of patients having had a PAH flare (25%). PAH is the main cause of mortality in MCTD, with proportions varying according to the literature. This association between connective tissue disease and PAH has already been described in the adult Afro-Caribbean population of Martinique, with a higher frequency than in other series [40]. The proportion in our cohort was higher than that described in the few pediatric series mostly Caucasian [3, 26, 27, 29, 38], but consistent with a long-term follow-up cohort of patients treated for MCTD [5].

One of the strengths of this study is its multicenter nature, with the participation of all the referring pediatricians in the FWI. Our methodology enabled us to identify patients and their therapies by referring to the national registry for rare diseases, as well the registries of local clinicians and by exploring computerized hospital archives and PMSI. This led to exhaustive identification of patients, and we crosschecked data from multiple sources to minimize the loss of patients and data. Secondly, we were able to study the initial and evolving characteristics of this Afro-descendant population in a country with a high standard of care and a universal, free healthcare system with limited bias related to social and economic status. Thirdly, the average length of follow-up was long, allowing us to obtain data from patients in their young adulthood.

This study had several limitations. First, it was retrospective and included a relatively small sample size; however, our cohort was one of the largest focusing on pediatric MCTD and OAS. Second, our data suffer from a lack of validated disease activity and damage scores for MCTD and OAS. Third, the incidence in our study may have been underestimated, due to the bias of retrospective studies on rare diseases covering a long period. Fourth, there was also a potential for recruitment bias, as our methodology mainly identified patients who required hospitalization. Nevertheless, most patients with MCTD and/or OAS generally benefit from at least one hospitalization for exploratory investigations. Despite these limitations, given the rarity of MCTD and OAS in children and the lack of previous description of patients of African ancestry, our multicenter study will contribute to the knowledge of these complex autoimmune diseases in children.

Overall, this is the first pediatric cohort of MCTD and OAS in Afro-descendant patients treated in a country with a high standard of care with longitudinal follow-up data. The clinical phenotypes were predominantly lupus and systemic sclerosis. The clinical signs and evolution profile did not differ between MCTD and OAS. The main complication causing morbidity was pulmonary arterial hypertension, which was more frequent in our cohort.

### Supplementary Information

**Additional file 1: Supplementary Figure 1.** Flowchart of the study.

## Data Availability

All data generated or analyzed during this study are included in this published article and tables.

## Abbreviations

OAS: Overlap autoimmune syndromes
MCTD: Mixed connective tissue disease
SLE: Systemic lupus erythematosus
SSc: Systemic sclerosis
DM/AM: Dermatomyositis/autoimmune myositis
JIA: Juvenile idiopathic arthritis
GGS: Gougerot-Sjögren syndrome
FWI: French West Indies
PMSI: French Medicalization of Information Systems Program
ACR: American College of Rheumatology
PAH: Pulmonary arterial hypertension
