# Supplementary material for: Longitudinal follow-up of mixed connective tissue disease and overlapping autoimmune diseases of childhood onset in the Afro-descendant population of the French West Indies
Source: Pediatr Rheumatol Online J. 2024 Jan 11;22:13. doi: 10.1186/s12969-023-00951-3 (PMC10785358; doi:10.1186/s12969-023-00951-3)
Supplement: Supplementary file 1 — Additional file 1: Supplementary Figure 1. Flowchart of the study. [file 12969_2023_951_MOESM1_ESM.docx]

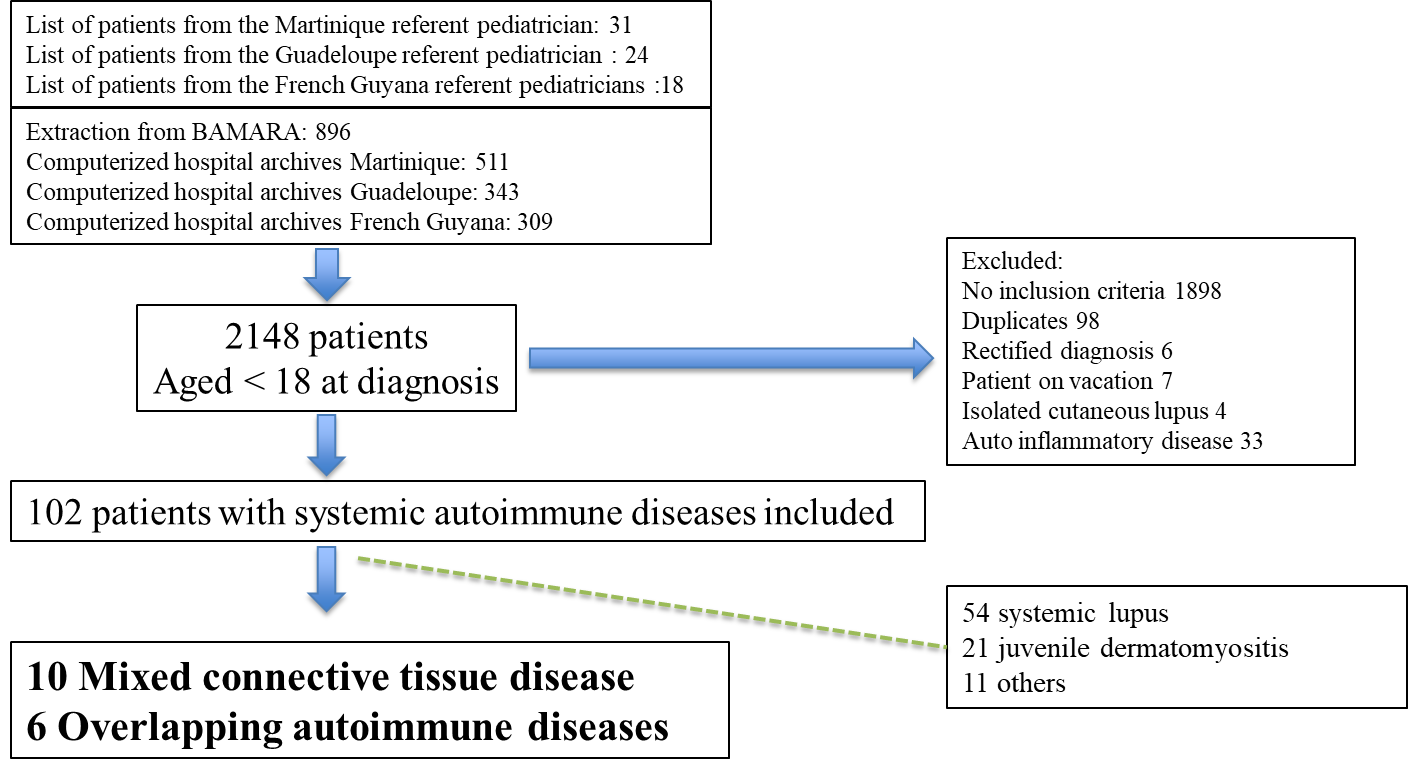


**Supplementary Figure 1:** Flowchart of the study.

*BAMARA stands for French National registry for rare diseases.*
